# Custom inspection on drunk driving in traffic accident injury

**DOI:** 10.1186/1471-227X-12-S1-A5

**Published:** 2012-12-18

**Authors:** MENG Fanshan, WANG Shuhou, ZHAO Yulan

**Affiliations:** 1 Emergency Department, the 88th Hospital of PLA, Taian 271000, Shandong, China

## Objective

To investigate the effect of custom inspection on drunk driving in traffic accident injury.

## Methods

In order to find out the effect of the strict check on drunk driving in traffic accident injury, we investigated the patients with traffic accident injury, who were sent to our hospital during the period from August to December, 2009, and compared them with those in the same period of 2007 and 2008.

## Results

The numbers of patients with traffic accident injury and the times of ambulance turnout in 2009 were fewer than those in 2007 and 2008. The percentage of critical patients and the number of car related traffic injury were decreased too.

## Conclusion

Custom inspection on drunk driving can effectively decrease the number of traffic accident injury and lower the percentage of critical patients.

